# Analysis of HLA-G long-read genomic sequences in mother–offspring pairs with preeclampsia

**DOI:** 10.1038/s41598-020-77081-3

**Published:** 2020-11-18

**Authors:** Ayako Nishizawa, Kazuki Kumada, Keiko Tateno, Maiko Wagata, Sakae Saito, Fumiki Katsuoka, Satoshi Mizuno, Soichi Ogishima, Masayuki Yamamoto, Jun Yasuda, Junichi Sugawara

**Affiliations:** 1 Division of Feto-Maternal Medical Science, Tohoku Medical Megabank Organization (ToMMo), Tohoku University, 2-1 Seiryo-machi, Aoba-ku, Sendai, 980-8573 Japan; 2 Tohoku University Graduate School of Medicine, Tohoku University, 2-1 Seiryo-machi, Aoba-ku, Sendai, 980-8575 Japan; 3 Department of Biobank, ToMMo, Tohoku University, 2-1 Seiryo-machi, Aoba-ku, Sendai, 980-8573 Japan; 4 The Group of Genome Sequence Analysis, ToMMo, Tohoku University, 2-1 Seiryo-machi, Aoba-ku, Sendai, 980-8573 Japan; 5 Department of Informatics for Genomic Medicine, Group of Integrated Database Systems, ToMMo, Tohoku University, 2-1 Seiryo-machi, Aoba-ku, Sendai, 980-8573 Japan; 6 Division of Molecular and Cellular Oncology, Miyagi Cancer Center Research Institute, 47-1 Nodayama Medeshima-Shiode, Natori, Miyagi 981-1293 Japan

**Keywords:** Genetics, Molecular medicine

## Abstract

Preeclampsia is a pregnancy-induced disorder that is characterized by hypertension and is a leading cause of perinatal and maternal–fetal morbidity and mortality. HLA-G is thought to play important roles in maternal–fetal immune tolerance, and the associations between HLA-G gene polymorphisms and the onset of pregnancy-related diseases have been explored extensively. Because contiguous genomic sequencing is difficult, the association between the HLA-G genotype and preeclampsia onset is controversial. In this study, genomic sequences of the HLA-G region (5.2 kb) from 31 pairs of mother–offspring genomic DNA samples (18 pairs from normal pregnancies/births and 13 from preeclampsia births) were obtained by single-molecule real-time sequencing using the PacBio RS II platform. The HLA-G alleles identified in our cohort matched seven known HLA-G alleles, but we also identified two new HLA-G alleles at the fourth-field resolution and compared them with nucleotide sequences from a public database that consisted of coding sequences that cover the 3.1-kb HLA-G gene span. Intriguingly, a potential association between preeclampsia onset and the poly T stretch within the downstream region of the HLA-G*01:01:01:01 allele was found. Our study suggests that long-read sequencing of HLA-G will provide clues for characterizing HLA-G variants that are involved in the pathophysiology of preeclampsia.

## Introduction

Preeclampsia (PE) is a pregnancy-induced condition that is characterized by hypertension and is a leading cause of perinatal and maternal–fetal morbidity and mortality. Recently, epidemiological studies have suggested that expectant mothers who are affected by PE have a higher risk of cardiovascular diseases, including hypertension, in later life^1–3^. Although extensive studies have been conducted, the etiology of PE remains unknown. However, multiple factors have been implicated, including endothelial^4,5^, immunological^6^, and genetic factors^7,8^. It has been suggested that an inadequate maternal immunological response to the semiallogenic fetus and consequently abnormal placentation could cause PE. The induction of immune tolerance to the fetus might be critical for the mother, and histocompatibility is expected to play an important role in this immune tolerance.

HLA-G, which is expressed in the extravillous trophoblasts of the placenta, is believed to play an important role in the establishment of maternal–fetal immune tolerance, and associations between HLA-G gene polymorphisms and PE have been suggested^9^. Fournel et al. suggested that the soluble form of the HLA-G protein secreted from extravillous trophoblasts could protect the fetus from the mother’s immunity^10^. Similarly, Fuzzi et al. suggested that only embryos that produce soluble HLA-G can survive after in vitro fertilization^11^. With regard to the relationship between HLA-G expression and PE, Farina et al. showed that mRNA expression of HLA-G was decreased in PE patients compared with healthy controls^12^. These studies suggest that soluble HLA-G plays a critical role in the induction of immune tolerance in the fetus.

The genetic relationship between HLA-G polymorphisms and PE, however, has not been clearly demonstrated. Hylenius et al. reported that paternal inheritance of a 14-bp deletion/insertion in HLA-G can be important for PE onset^13^. However, several follow-up studies failed to support the findings of Hylenius et al.^14–17^. Among those studies, Larsen et al. found that the 14-bp deletion/insertion polymorphism (rs371194629) accompanying other polymorphisms in exon 8 (rs1710, rs1063320, rs9380142, and rs1610696) was associated with PE in the primipara when the fetus was homozygous for the insertion^18^. The results by Larsen et al. indicate the importance of contiguous haplotype data for investigation of the association between HLA-G and PE.

Previous HLA-G genotyping studies of patients with PE have exhibited controversial results. Genotyping of HLA genes is quite difficult because of its complex structure. Tadaka et al. discussed obvious discordance in the allele frequencies at the HLA loci between the GnomAD^19^ and 3.5KJPN databases (a whole-genome reference panel for the Japanese population^20^). A solution to the issue of uncertain genotypes at the HLA loci might be long-read sequencing^21^. The length of the HLA-G locus (5.2 kb) is within the PCR amplification range if appropriate primer sets are designed^20^. This finding might be useful for identifying unique, nonpolymorphic regions that flank the HLA-G locus. Therefore, the sequencing of PCR amplicons spanning the whole HLA-G locus using long-read sequencers is a feasible approach for population genetic studies of the association between PE and HLA-G variants.

In the present study, we first analyzed full‐length consensus sequences of the HLA-G locus in mother–offspring pairs with complications of PE and healthy pregnancies using single-molecule real-time sequencing on the PacBio RS II instrument. Additionally, we investigated PE-related distributions of HLA-G alleles and the compatibility of HLA-G genotypes in mother–offspring pairs and explored new alleles of the HLA-G locus.

## Results

### Determination of the sequences of the HLA-G region in 31 mother–offspring pairs

**Figure 1:**
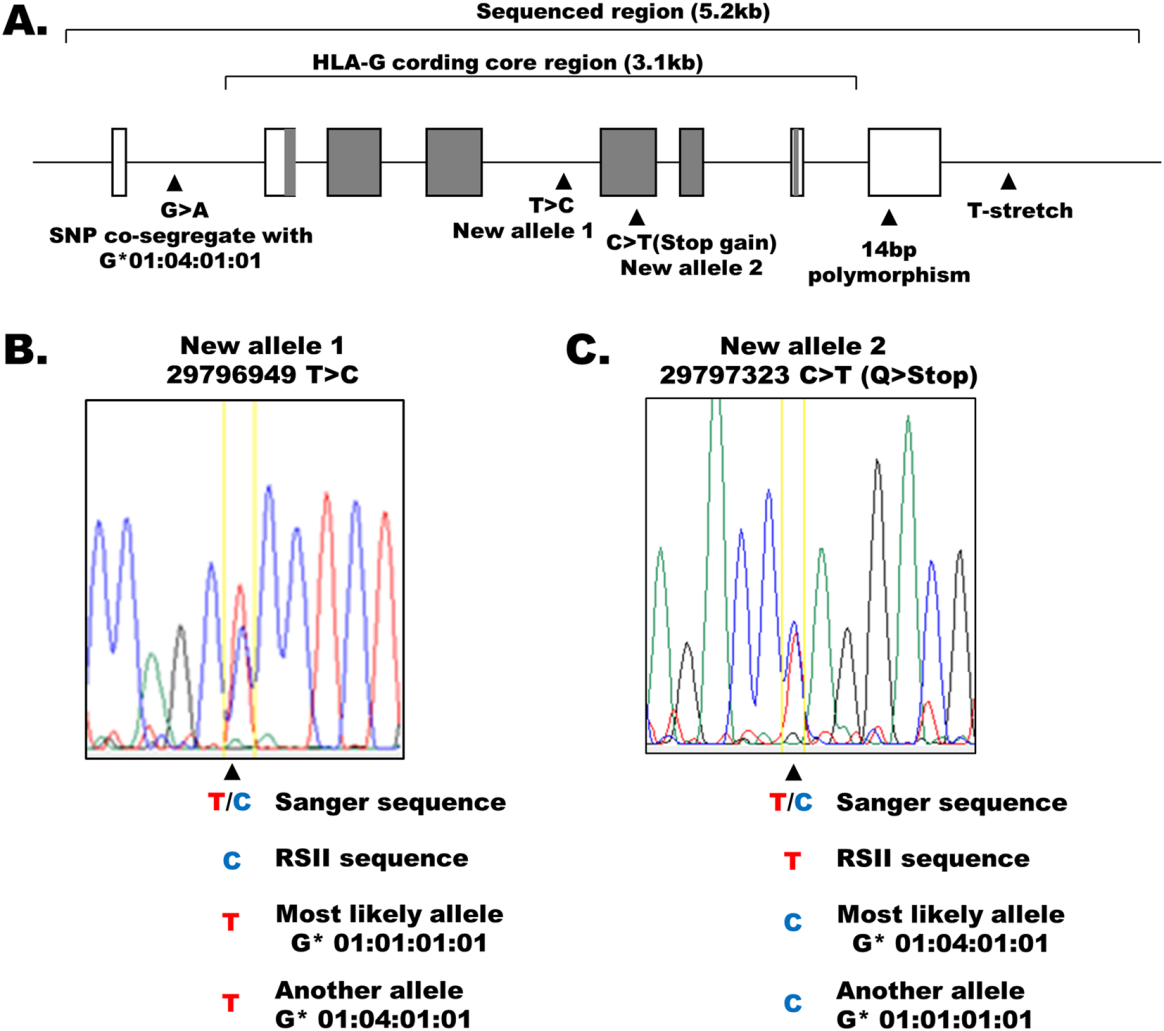
Novel alleles of the HLA-G core region. (**A**) Schematic of the HLA-G core region. White and gray rectangles indicate noncoding and coding exons, respectively. Lines that connect the rectangles indicate introns. The exon widths are proportional to the actual sizes, but the intron lengths are not. The HLA-G core region (defined based on the known HLA-G alleles registered in the IPD-IMGT/HLA database: https://www.ebi.ac.uk/ipd/imgt/hla/) and the region analyzed in the present study are indicated by the brackets above the exons/introns. The polymorphisms analyzed in this study are indicated by triangles and short descriptions. (**B**,**C**) Fluorographs of Sanger sequencing performed to verify the novel HLA-G core region alleles, with sense strand reads shown.

To elucidate the association between the HLA-G genotype and PE etiology, we determined the alleles of the HLA-G locus in a total of 31 mother-fetus pairs; 18 consisted of mothers with PE and 13 consisted of normal pregnancies/births and served as controls. The clinical characteristics of the mothers and infants are shown in Table 1. We amplified a 5.2 kb genomic region that contained the entire HLA-G gene by PCR in 62 genomic DNA samples and sequenced the amplicons using the PacBio RS II long-read sequencer to construct de novo consensus sequences of the amplified HLA-G region (Fig. 1A). We defined the HLA-G core region based on the longest known HLA-G alleles registered in the IPD-IMGT/HLA database (https://www.ebi.ac.uk/ipd/imgt/hla/). After filtration according to the number of subreads of a consensus sequence, we obtained one or two consensus sequences for each sample, which likely corresponded to homozygotes or heterozygotes, respectively (see “Methods” section for details).

**Table 1:** Summary of the study cases.

	Control (n = 13)		PE (n = 18)		p-value^a^
	Mean (%)	SD	Mean (%)	SD	
**Maternal age (years)**					
Mean, SD	30.9	3.2	31.2	5.8	0.56
**Parity (primipara)**					
%	30.7		38.9		0.64^b^
**Prepregnancy BMI**					
Mean, SD	22	4.43	22	3.15	0.5
**Gestational age at delivery (weeks)**					
Mean, SD	39.3	0.95	37.3	1.36	< 0.0001
**Birth weight (g)**					
Mean, SD	3082.6	402.8	2600.1	477.6	0.0034

*SD* standard deviation.

^a^Student t-test was performed unless otherwise indicated.

^b^Chi-square test.

The nucleotide sequences of the whole coding exons, introns, and flanking untranslated regions of HLA-G, which span 3.1 kb, are publicly available (ftp://ftp.ebi.ac.uk/pub/databases/ipd/imgt/hla/fasta/), and our amplicons covered the entire region. We compared the obtained PacBio consensus sequences with the sequences of known HLA-G alleles by a BLAST search. Most of the consensus sequences were consistent with one of seven major HLA-G alleles; however, eight PacBio consensus sequences were inconsistent with any known HLA-G alleles (Supplementary Table 1). One consensus sequence, C5_1516_2, could be similar to two different known HLA-G alleles (G*01:04:01:01 or G*01:01:02:01), and as a result, we compared the consensus sequence with these two HLA-G alleles (Supplementary Table 1). Considering the low accuracy of our long-read sequencing results, which might be caused by nested PCR, we confirmed the sequence of each mismatched site in these inconsistent sequences by Sanger sequencing (see “Methods” section for details). All of the mismatches, except for two, were corrected to the sequences of known alleles (Supplementary Table 1). The sites that were missequenced by the PacBio RSII were insertions or deletions of one or two residues in homopolymers (4–7 bp in length). In particular, at intron 7, there is a palindromic short stretch of homopolymers (‘CCCCGGGGGG’), and all of the errors in our PacBio sequencing in intron 7 occurred in the palindromic stretch. Similarly, the palindromic repeat in exon 2 (‘GGGGGCCC’) also showed a nucleotide deletion in two cases. These results indicate that only a few nucleotide errors occurred within short homopolymers by PacBio amplicon sequencing with nested PCR.

### Identification of novel alleles of the 3.1 kb HLA-G core region in our cohort

For the two PacBio consensus sequences that were inconsistent with the known HLA-G sequences, the Sanger sequencing results corresponded to the PacBio consensus data (Supplementary Table 1), which indicates that the inconsistency was because the HLA-G sequences are novel rather than the result of errors in the PacBio consensus sequences. Each of the two new alleles had one known additional single nucleotide polymorphism (SNP), rs1464043200 and rs144753960, relative to the known alleles that they resembled the most (Fig. 1B), G*01:01:01:01 and G*01:04:01:01, respectively (Supplementary Table 2). We searched the GenBank database using BLAST^22^ and found no sequences that were identical to either new allele (data not shown). Heritability was not confirmed for either new allele in our collection.

### HLA-G alleles and variants in the Japanese population

The distribution of the HLA-G alleles in the 18 PE and 13 control (i.e., normal pregnancies/births) mother–offspring pairs is shown in Table 2. Because the SMRT analysis software merges the same sequences into one consensus sequence, the homozygous alleles will appear as one sequence in an individual. Hence, the alleles with a single consensus sequence were counted as two of the same allele (i.e., homozygous), and a total of 124 alleles were obtained from the 31 mother–offspring pairs. Comparison of the determined alleles between each mother and her offspring indicated that in all pairs, the mother and her offspring shared at least one allele, which demonstrates that all mother–offspring pairs in our study can be biologically related.

**Table 2:** Distribution of HLA-G alleles in preeclampsia and healthy mother–offspring pairs.

HLA-G	14 bp	PE				Control				Total	p-value
		Mother	Offspring	Sum	Inherited	Mother	Offspring	Sum	Inherited		
01:01:01:01	Del	13	13	26	7*	7	9	16	4	42	0.569
New allele 1	Del	1	0	1	0	0	0	0	0	1	NA
01:01:02:01	+	7	5	12	4	3	6	9	3	21	1
01:01:03:03	+	4	2	6	2	4	0	4	0	10	1
01:03:01:02	+	1	1	2	1	0	0	0	0	2	0.509
01:04:01:01	Del	9	10	19	3*	10	8	18	4	37	0.329
New allele 2	Del	0	1	1	0	0	0	0	0	1	NA
01:04:01:02	Del	1	4	5	0	1	2	3	1	8	1
01:06:01:01	+	0	0	0	0	1	1	2	1	2	0.174

*Del* deletion, *NA* not analyzed.

*One mother–offspring pair showed the same genotype.

In our cohort, G*01:01:01:01 and G*01:04:01:01 were the most common alleles, accounting for 33.9% (42/124) and 29.8% (37/124) of all determined alleles, respectively. The data were consistent with previous reports that G*01:01:01 and G*01:04 are the most common alleles in the Japanese population^23,24^, although the HLA-G alleles of previous studies were limited to second- or third-field resolution, whereas our results were expanded to fourth-field resolution. The distribution of the HLA-G alleles was not significantly different between the PE and control mother–offspring pairs (Table 2), which suggests that known single polymorphic sites within the HLA-G core region do not affect PE onset.

Recent progress in identifying whole-genome variants in the general population using short-read resequencing data enabled us to compare polymorphisms in the HLA-G region with those in much larger datasets of the general population. A comparison of our results with previously reported minor allele frequencies in the HLA-G core region in the East Asian population, using the gnomAD database (gnomAD-EAS)^19^, and in the Japanese population, using the Tohoku Medical Megabank Organization (ToMMo-4.7KJPN)^25^, showed that our frequencies for each SNP and short insertion/deletion (indel) were highly consistent with those large-scale data (R = 0.964 and R = 0.996 for GnomAD-EAS and ToMMo-4.7KJPN, respectively) and confirmed the accuracy of our sequencing results (Supplementary Fig. 1).

### The lack of an association of the 14-bp indel polymorphism in HLA-G exon 8 with PE

As mentioned above, the 14-bp indel in exon 8 of the HLA-G gene (rs371194629, chr6: 29,798,581 in hg19) is controversial in terms of its association with PE. This indel is located outside of the well-characterized 3.1 kb HLA-G core region, and the insertion is conserved in the chimpanzee genome (panTro6/chr6: 29,349,721–29,349,735). The relationship between HLA-G alleles and the 14-bp indel was evaluated by long-read de novo sequencing using PacBio RS II. The G*01:01:02:01, G*01:01:03:03, G*01:03:01:02, and G*01:06:01:01 alleles contain the 14-bp insertion, whereas the other alleles do not, which is the same as in the human reference genome sequence (14-bp deletion, Table 2). These contiguous data correspond to those obtained by Larsen et al.^18^. There was no significant difference in the allelic frequencies of the 14-bp indel between PE and healthy mother–offspring pairs in our cohort (p > 0.05, Fisher’s exact test, Table 2).

### SNPs showing new cosegregation with a certain HLA-G allele

We also evaluated potential associations of the variants outside of the 3.1 kb HLA-G core region with PE onset (Supplementary Table 3). An SNP was found upstream of the core region (rs17875394, in intron 1), and its relationship with HLA-G haplotypes was unknown. In our collection, this variant was only cosegregated with G*01:04:01:01. Of the 38 G*01:04:01:01 alleles (including one new allele), 14 had the A allele in our cohort. As expected, the frequency observed in this study (14 of 93 total chromosomes, 0.150) was similar to that in 4.7KJPN; the minor allele frequency of the polymorphism was 0.1488 in 4.7KJPN and 0.0855 in GnomAD-EAS. The polymorphism was not associated with PE onset.

### An unexpected pattern of the length of the downstream poly T stretch

Comparison of the allele frequencies of SNPs or known indels in the HLA-G region observed in our biased collection as well as in larger general population data suggested that there would be few differences in the frequencies of known HLA-G alleles between the PE and healthy mother–offspring pairs in our dataset. In fact, there was no significant difference in the HLA-G allele frequencies between the groups (Table 2). In addition, any combination of two HLA-G alleles in an individual did not show any significant association with PE onset (data not shown). These results suggest that previously known HLA-G alleles might not be related to the etiology of PE.

When evaluating the fidelity of the PacBio reads of very low complexity sequences, such as homopolymers, we encountered a long poly T stretch that was located downstream of HLA-G (hg19/chr6: 29,799,206–29,799,226). The length of the poly T stretch in hg19 is 21 bp, and the corresponding length in the chimpanzee genome is 24 bp. The stretch is polymorphic, and multiple alleles have been reported in the WebSTR database (https://webstr.ucsc.edu/)^26^ as well as the 4.7KJPN database. The length of the stretches is 17–43 bp in our collection, 13–38 bp in 4.7KJPN, and 17–50 bp in the WebSTR database. Even though the range of the lengths of the T stretches we observed is within the ranges of the previous studies, we further checked the differences in the T stretch length between mother and offspring alleles that would be shared between the two and found that the differences would be approximately 0.8 to 2.8 among those cases (Supplementary Table 4). As expected, longer T stretches show larger differences, and the average intergeneration differences in T stretches with lengths less than 26 or more than 35 are 0.8 or 2.8, respectively (Supplementary Table 4). These results indicate that the two-stage PCR did not largely affect the length of the T stretches in our samples. The poly T stretch does not appear to be related to eQTL in the GTEx project database^27^, and placenta data are not included in the dataset^28^. Considering the 10–15% contribution of short tandem repeat variance to eQTL^27^, the poly T stretch might play some role in regulating HLA-G expression in the placenta.

**Figure 2:**
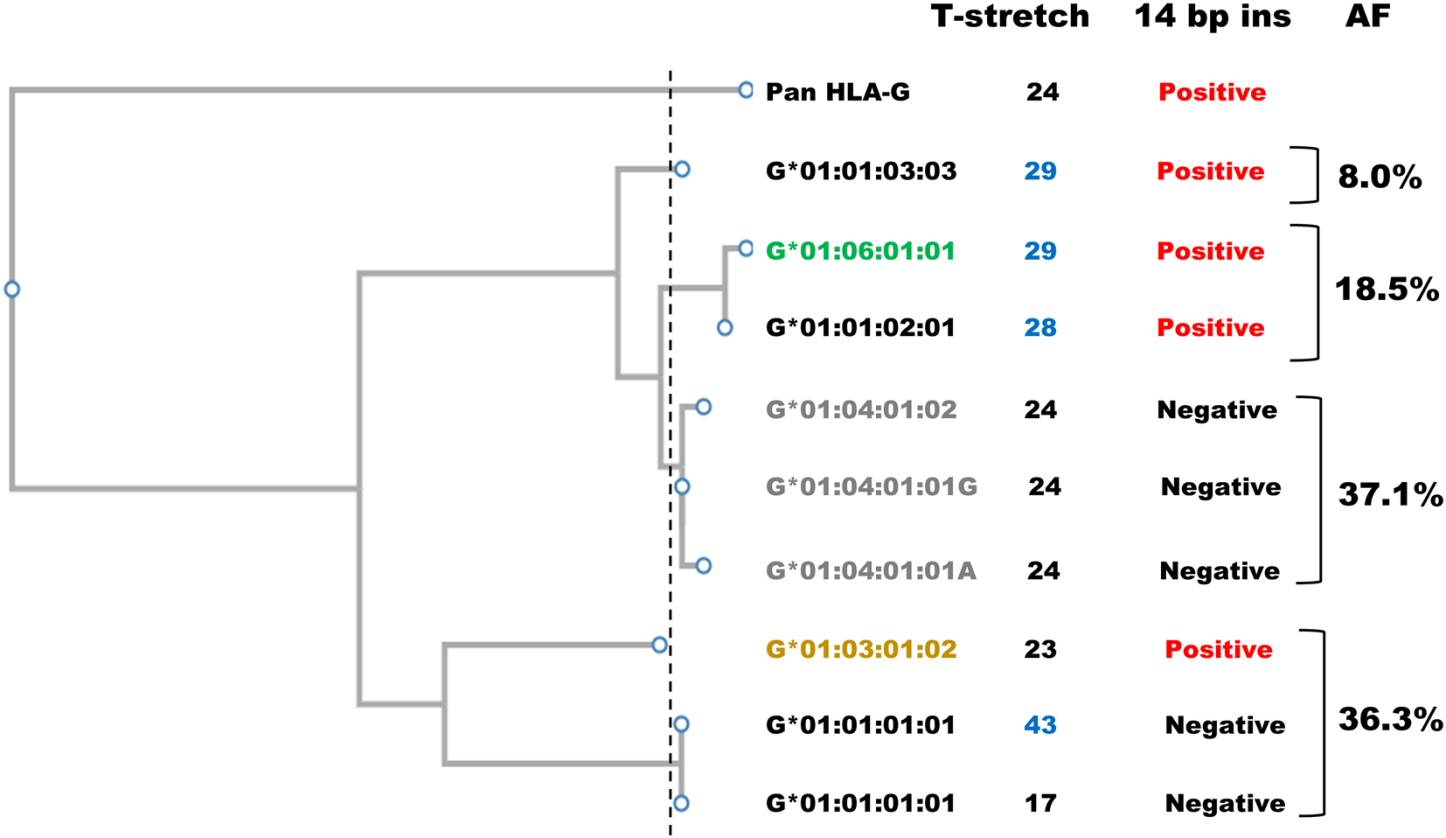
Dendrogram of the molecular evolution of HLA-G. The seven major haplotypes of HLA-G found in this study and the corresponding Pan HLA-G sequences (panTro6: chr6: 29,345,638–29,350,879) were analyzed using ClustalW software. We used two G*01:01:01:01 alleles with different T stretch lengths (17 and 43 bp) and two G*01:04:01:01 alleles based on the variant rs17875394. The allelotypes with second field resolution are indicated with different colors: 01:01 as black, 01:03 as orange, 01:04 as gray, and 01:06 as light green. The vertical distance indicates the difference between the alleles, and the horizontal distance indicates the relative timing of the separation among the alleles. The broken line indicates the categories used for the allele frequencies. The presence or absence of the 14-bp insertion is indicated as “positive” in red and “negative” in black on the right side of the panel. The nucleotide lengths of the T stretch (figures longer than 25 are indicated in blue) and the cumulative allele frequencies of each category in our data set are shown on the right side of the panel.

The WebSTR data indicate that multiple HLA-G alleles are present in several ethnicities. Thus, we evaluated whether any diversity in the length of the poly T stretch was present in our sequencing collection and the relationship of the poly T stretch length with known HLA-G alleles. We constructed a phylogenetic tree of the HLA-G alleles (Fig. 2, see legend). The structural variations, the 14-bp indel in the 3′-untranslated region and the length of the poly T stretch in the downstream region showed distinct branching. Intriguingly, the presence versus absence of the 14-bp indel did not match the branching pattern of the dendrogram drawn based on the information on the HLA-G core region or the allele nomenclature. For example, G*01:01:01:01 and G*01:03:01:02 are located in the same branch, but the presence of the 14-bp insertion was discordant between them. Similarly, three different G*01:01 alleles were found in our dataset, but only G*01:01:01:01 lacked the 14-bp insertion. This finding suggests that a recombination hot spot might exist between the HLA-G core and the downstream regions, including the 14-bp indel and polymorphic T stretch.

### Relationship between the length of the T stretch downstream of HLA-G and PE onset

**Figure 3:**
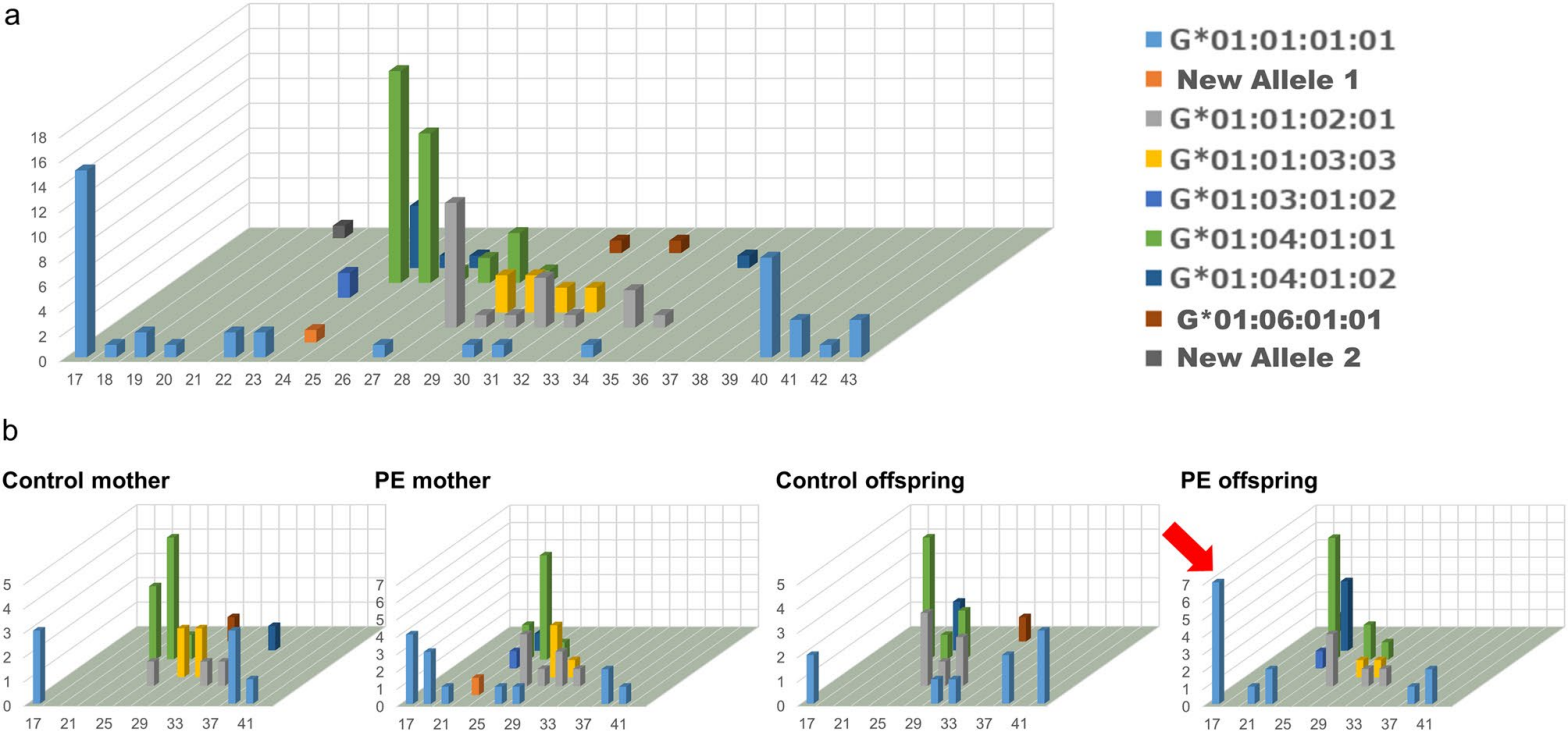
Allelic variations in the nucleotide length of the T stretch downstream of HLA-G. (**a**) Three-dimensional bar graph of the poly T stretch length. The x-axis indicates the nucleotide length of the poly T stretch, and the y-axis indicates the number of chromosomes observed in this study. Chromosomes shared between mother and offspring were counted as one. The z-axis indicates the HLA-G alleles observed in this study. The indexes are shown on the right side of the bar graph. (**b**) Three-dimensional bar graphs of the T stretch according to the presence or absence of PE and the generation (mother vs. offspring). The statistically significant data for PE offspring (the shortest T stretch associated with G*01:01:01:01) in the rightmost graph are indicated with a red arrow.

There are no reports that describe the association between the poly T stretch downstream of HLA-G and PE onset. Figure 3 shows the distribution of the T stretch length among all known HLA-G alleles. The 01:04:01:01 allele had a peak T stretch length of 24 bp and was monophasic. As expected, the most prevalent allele, G*01:01:01:01, showed a wide range of lengths (from 17 to 46 bp), and interestingly, the length had a biphasic distribution with two peaks, at 19 and 40 bp (Fig. 3a). We focused on this allele because it had the greatest variability in the T stretch length. Because the length of the corresponding T stretch in the chimpanzee genome is 24 bp, we classified the alleles as shorter (< 25 bp) or longer (≥ 25 bp) and compared the distribution between the healthy and PE mother–offspring pairs.

Interestingly, G*01:01:01:01 had a significantly longer T stretch in the healthy control offspring than in the PE offspring (p = 0.0274, Fisher’s exact test) (Fig. 3b, Table 3). In addition, the length of the T stretch inherited from mother to offspring was obviously shorter in the PE offspring only for G*01:01:01:01 compared with the other HLA-G alleles, which suggests that shorter T stretches in G*01:01:01:01 in the fetus could promote PE in the mother (Supplementary Fig. 2), although we did not detect any suspicious variants in G*01:01:01:01 with a shorter poly T stretch in our dataset.

**Table 3:** Comparison of the length of the poly T-stretch in the G*01:01:01:01 allele between healthy and preeclampsia mother–offspring pairs.

G*01:01:01:01	Short	Long	p-value	Odds ratio (95% confidence interval)
	≤ 25 bp	> 25 bp		
**Offspring**				
PE	10	3	0.0274	11.67 (1.53–89.12)
Healthy	2	7		
**Mother**				
PE	8	5	0.6424	2.13 (0.33–13.81)
Healthy	3	4		

## Discussion

Using single-molecule real-time sequencing on the PacBio RS II instrument, we obtained full‐length consensus sequences of HLA-G loci in 18 PE and 13 healthy mother–offspring pairs. The PacBio long-read sequencing data corresponded well to short-read resequencing population data. In addition, two new alleles of HLA-G were identified in addition to seven well-known HLA-G alleles. Moreover, a long T stretch downstream of HLA-G outside of the commonly characterized 3.1 kb region was found. The lengths of the stretches showed divergence (from 17 to 43 bp). Interestingly, the shorter poly T stretch (< 25 bp) in the HLA-G*01:01:01:01 allele, the most prevalent HLA-G allele, in the offspring appears to be associated with the onset of maternal PE in a dominant manner.

The PacBio sequencing results corresponded well to those of previous studies. For example, the minor allele frequencies of the HLA-G region were similar to those in the 4.7KJPN and GnomAD EAS populations. This finding suggests that the PCR amplicon sequencing approach can be used together with PacBio sequencing, whose high accuracy is shown in human whole genome sequencing^29^. Based on the data shown in Supplementary Table 1, 8 amplicon sequencing datasets showed 16 errors in consensus sequences of 3.1 kb. Therefore, the misincorporation rate would be 1 in 1.55 kb. Therefore, artifacts introduced by PCR seem to be negligible in our study although our approach should be interpreted in caution because we could not verify all the sequencing data by other means. Thus far, we observed only one possible chimeric consensus sequence in our dataset, which indicates that the new alleles are valid alleles, not chimeric. We might miss new alleles among the filtered low-quality consensus sequences. However, it is unlikely that the number of accumulated reads of the homozygous allele is nearly twice that of one allele in heterozygous samples (data not shown). The new alleles found in our collection indicate that there are many uncharacterized HLA-G alleles in the human population, and some of these alleles might have a strong association with PE onset.

The soluble form of HLA-G could play a role in the induction of immune tolerance mediated by CD8+ T cells^9^. However, we did not find any direct genetic evidence that pertained to the function of soluble HLA-G in our collection. A novel allele that contained the stop-gain SNP rs144753960, observed in a PE mother (Table 2), was consistent with previous studies that demonstrated that PE etiology is correlated with decreased expression of HLA-G^30^. However, our sample size might not be sufficient to achieve a significant result with a strict statistical correction.

The length of the poly T stretch located downstream of the HLA-G gene is unlikely to affect the HLA-G protein structure directly. G*01:01:01:01 is the most common allele of HLA-G in humans, and the biphasic distribution of the length of the poly T stretch downstream of G*01:01:01:01 is a new finding (Fig. 3a). The evolutionary mechanism of this biphasic distribution is unknown. According to Willems et al., short tandem repeats of 2–6 bp tend to show a monophasic, symmetric distribution of the repeat length^26^. Roy-Engel et al. reported that active *Alu* elements have a longer poly A stretch, and several *Alu* subtypes showed a biphasic distribution of the A stretch^31^. Hence, mononucleotide repeats could have a different length distribution than those of other short tandem repeats, although the HLA-G downstream poly T stretch does not appear to be related to *Alu* repeats.

The molecular mechanisms of the association between the length of poly T stretches downstream of HLA-G*01:01:01:01 and the onset of PE are unknown. Possibly, the shorter T stretch allele of G*01:01:01:01 could be linked to the further upstream or downstream cis-regulatory elements that cause downregulation of soluble HLA-G expression. It will be necessary to characterize the longer region of the HLA-G flanking sequences to address this issue, and most likely, whole genome DNA would be necessary to obtain continuous sequence data from the T stretches to the possible cis-elements.

Several limitations should be considered in this study. First, the results of the present study exhibited only weak statistical significance based on the small sample size. We should validate the results of the present study in other data sets with larger sample sizes. Second, because a minimum amount of genomic DNA is available, we had to perform two rounds of PCR amplification for our analysis, and the possible PCR artifacts could cause false positives or negatives. In particular, the simple repeats are difficult to be precisely sequenced by all amplification and sequencing technologies and sequencing errors are often reported as deletions.

Nonetheless, our results suggest that the HLA-G*01:01:01:01 allele with a shorter downstream poly T stretch plays a role in PE onset. HLA-G is expressed mainly in trophoblasts of the placenta. The finding that the G*01:01:01:01:01 allele with a shorter poly T stretch in offspring was more strongly associated with PE does not refute our hypothesis that G*01:01:01:01 with a shorter poly T stretch is associated with PE. Considering the prevalence of G*01:01:01:01 in the population, the length of the poly T stretch in this allele might be a good predictor of PE, although it could be difficult to establish a method to evaluate this specific allele. Further research, including functional studies of HLA-G polymorphisms and the poly T stretch, would provide new insights into the etiology of PE.

## Methods

### DNA samples

Subjects were obtained from the Birth and Three-Generation Cohort Study established by the Tohoku Medical Megabank Project^32^. Pregnant mothers were first recruited from obstetrics clinics, and their husbands and parents were then recruited; their consent to evaluate their infants at birth was obtained. More than 20,000 families participated in this cohort study. Among the participants, PE-affected and healthy mother–offspring pairs were diagnosed using a clinical database in the Tohoku Medical Megabank Organization. PE was diagnosed in accordance with the American Congress of Obstetricians and Gynecologist criteria^33^. PE was determined according to the following conditions using blood pressure and urinary paper tests at each perinatal checkup. PE is systolic blood pressure ≥ 140 mmHg or diastolic blood pressure ≥ 90 mmHg after 20 weeks of gestational age with proteinuria ≥ 1+. Control pairs were defined as healthy mother–offspring pairs without any maternal or fetal/neonatal complications. The number of analyzed samples was determined by calculations based on the cost requirement for long-read whole genome sequencing. Consequently, a total of 31 pairs of maternal and umbilical cord blood genomic DNA samples, 18 from PE-affected and 13 from healthy pairs, were randomly selected from the Birth and Three-Generation cohort project of the Tohoku Medical Megabank Organization^32^. Written informed consent was obtained from every subject. The research protocols of the present study were reviewed and approved by the Ethics Committee of ToMMo (2014-0002-2, 2019-4-006, and 2019-4-035). This study was conducted in accordance with the Declaration of Helsinki. Genomic DNA was extracted from whole peripheral blood (mothers) or umbilical cord blood (offspring) as described in^34^.

### Long-read amplicon sequencing of the HLA-G locus by PCR

We attempted to sequence the whole region of the HLA-G gene, including the promoter region (5.2 kb). In general, HLA genes are rich in polymorphisms and homologous to each other. Therefore, it is difficult to obtain PCR primers for HLA genes that can be applied to all individuals of a large population. We employed Primer 3 software^35^ to design candidate PCR primer pairs for the HLA-G region after masking the repetitive sequences using RepeatMasker (4.0.7)^36^. To ensure successful PCR for as many samples as possible, we used a Japanese whole-genome reference panel (3.5KJPN version 2)^20^ to exclude the PCR primers whose five bases at the 3′ end overlap any polymorphic sites. Finally, we searched for potential off-target annealing sites of the primers using primer BLAST in the nr database (National Center for Biotechnology Information, https://www.ncbi.nlm.nih.gov/). The primer sequences are shown in Supplementary Table 5.

To obtain the template DNA fragment used for amplicon sequencing, two-step PCR was performed under the following conditions. Template DNA (20 ng human genomic DNA in the first step, 5 ng amplified DNA fragments in the second step) was added to 0.75 U PrimeSTAR GXL DNA polymerase (Takara Bio Inc., Shiga, Japan), 2 pmol/µl forward and reverse primers (Supplementary Table 5), and 10 nmol/µl dNTP mixture with 1× PrimeSTAR GXL Buffer (Mg2+ plus). The thermal cycling protocol, run on the Applied Biosystems ProFlex PCR System thermal cycler (Thermo Fisher Scientific, Waltham, MA), was as follows: initial denaturation step at 98 °C for 1 min, followed by 20 cycles of amplification at 98 °C for 10 s and at 65 °C for 15 s, and a final elongation step at 68 °C for 6 min. The purity and amount of the PCR product were checked by 1% agarose gel electrophoresis using 1× TAB with MUPID II (Takara Bio Inc.).

### Library construction and PacBio RS II sequencing

The library construction for PacBio RS II sequencing was performed following the manufacturer’s instructions (Pacific Biosciences, Menlo Park, CA). We have seven amplicon libraries per sample, including samples for other projects (data not shown). Eight samples were subjected to long-read sequencing in one SMRT cell (a total of 56 different amplicons/SMRT cell), and each amplicon library was adjusted to 0.083 pmol/SMRT cell. To distinguish individuals, we added barcodes during the second PCR step. Adjustment of the library DNA concentration was performed using Binding Calculator ver. 2.3.1.1. PacBio sequencing reactions were conducted using P6-C4 chemistry following the manufacturer’s protocol (Pacific Biosciences).

### Variant calling in PacBio amplicon sequencing data for HLA-G

The sequencing data for the HLA-G locus were analyzed using RS_Long_Amplicon_Analysis in SMRT analysis version 2.3.0 software (Pacific Biosciences). Because seven amplicons from eight samples were analyzed in an SMRT cell, we had to distinguish DNA barcodes using the option “Different on each end (paired)”. Consensus reads of the HLA-G amplicons were built using a minimum subread length of 5064 and a maximum number of subreads of 4000 after several trials. To filter out less reliable consensus reads, we employed filtering by setting the number of reads in a consensus to > 84. One consensus sequence that appeared to be a chimera of two known alleles was removed after manual inspection.

### Verification of long-read sequencing data

After comparison with known HLA-G sequences, there were 41 discordant bases among all 124 HLA-G consensus sequences obtained by PacBio. To verify the discrepancies, we employed cycle sequencing to detect single nucleotide differences, including deletions, and 10% polyacrylamide gel electrophoresis to detect longer nucleotide differences found within the 3′ poly T stretch of amplicons of the 01:01:01:01 allele. In both cases, we used the insert libraries from PacBio sequencing as PCR templates. The primers are shown in Supplementary Table 5. The PCR conditions for the template DNA and size separation on polyacrylamide gel electrophoresis were the same as those used in the second amplification step of the PacBio library construction. The Applied Biosystems ProFlex PCR System (Thermo Fisher Scientific) and Veriti 96-Well Thermal Cycler (Thermo Fisher Scientific) were used. Capillary sequencing was conducted using the Applied Biosystems 3500 XL sequencer (Thermo Fisher Scientific).

### Analyses of HLA-G allele sequences

A comparison between the known HLA-G alleles and PacBio consensus reads was performed using the BLAST command-line tool with the default options^22^. A dendrogram of the HLA-G alleles was drawn using ClustalW with the default options in PhyML (https://www.genome.jp/). Other statistical comparisons were performed using Fisher’s exact test. We considered the results to be statistically significant at p < 0.05.

## Supplementary information

Supplementary Information.
