# Supplementary material for: Analysis of HLA-G long-read genomic sequences in mother–offspring pairs with preeclampsia
Source: Sci Rep. 2020 Nov 18;10:20027. doi: 10.1038/s41598-020-77081-3 (PMC7675977; doi:10.1038/s41598-020-77081-3)

Supplementary information

Analysis of HLA-G long-read genomic sequences in mother-offspring pairs with preeclampsia

Ayako Nishizawa[1,2], Kazuki Kumada[3*], Keiko Tateno[4], Maiko Wagata[1, 2], Sakae Saito[2, 4], Fumiki Katsuoka[2, 4], Satoshi Mizuno[2,5], Soichi Ogishima[2,5], Masayuki Yamamoto[2, 4], Jun Yasuda[4, 6], and Junichi Sugawara[1, 2*]

[1] Division of Feto-Maternal Medical Science, Tohoku Medical Megabank Organization (ToMMo), Tohoku University, 2-1 Seiryo-machi, Aoba-ku, Sendai 980-8573, Japan,

[2] Tohoku University Graduate School of Medicine, Tohoku University, 2-1 Seiryo-machi, Aoba-ku, Sendai 980-8575, Japan,

[3] Department of Biobank, ToMMo, Tohoku University, 2-1 Seiryo-machi, Aoba-ku, Sendai 980-8573, Japan,

[4] The Group of Genome Sequence Analysis, ToMMo, Tohoku University, 2-1 Seiryo-machi, Aoba-ku, Sendai 980-8573, Japan,

[5] Department of Informatics for Genomic Medicine, Group of Integrated Database Systems, ToMMo, Tohoku University, 2-1 Seiryo-machi, Aoba-ku, Sendai 980-8573, Japan,

[6] Division of Molecular Carcinogenesis, Miyagi Cancer Center Research Institute, 47-1, Nodayama, Medeshima-shiote, Natori, Miyagi 981-1293, Japan.

*Corresponding authors: Junichi Sugawara and Kazuki Kumada

Junichi Sugawara, M.D., Ph.D.

Division of Feto-Maternal Medical Science, Tohoku Medical Megabank Organization (ToMMo), Tohoku University, 2-1 Seiryo-machi, Aoba-ku, Sendai 980-8573, Japan

E-mail: jsugawara@med.tohoku.ac.jp

Kazuki Kumada, Ph.D.

Department of Biobank, ToMMo, Tohoku University, 2-1 Seiryo-machi, Aoba-ku, Sendai 980-8573, Japan

E-mail: kazuki.kumada@megabank.tohoku.ac.jp

Supplementary table 1. List of verification of PacBio data with Sanger sequencing

Consensus sequence	Mother/Offspring	Most likely known allele (candidate)	Mismatched sites							Evaluation results
			hg19 position	IDP-IMGT position	Exon/Intron	Residue in candidate sequence	Residue in RII sequence	Residue in second allele	Sanger sequence	
C1_910 _2	Mother	01:01:03:03	29796822	1201	Intron 4	G	-	G	G/G	01:01:03:03
			29797202	1581	Exon 5	C	-	C	C/C	
			29797743	2112	Intron 6	T	-	T	T/T	
C1_1112 _2	Offspring	01:04:01:01	29798393	2772	Intron 7	G	-	G	G/G	01:04:01:01
C5_1516_2	Offspring	01:04:01:01	29797639	2018	Exon 6	C	T	C	C/T	01:01:02:01
			29798419	2798	Intron 7	G	A	G	G/A	
		01:01:02:01	29796327	706	Exon 4	T	C	C	C/T	
			29796376	755	Exon 4	C	A	A	C/A	
C8_34_2	Offspring	01:01:01:01	29795667	46	Exon 2	C	-	C	C/C	New allele 1
			29796949	1328	Intron 4	T	C	T	C/T	
			29798393	2772	Intron 7	G	-	G	G/G	
C2_1314_2	Mother	01:04:01:01	29798393	2772	Intron 7	G	-	G	G/G	01:04:01:01
C6_56_2	Mother	01:04:01:01	29797323	1702	Exon 5	C	T	C	C/T	New allele 2
C1_56_2	Mother	01:01:02:01	29795667	46	Exon 2	C	-	C	C/C	01:01:02:01
C7_1516_2	Offspring	01:04:01:01	29798387	2766	Intron 7	C	-	C	C/C	01:04:01:01

Supplementary table 2. Novel alleles of HLA-G core region in this study

	Most similar to	Position	Ref*	Alt	SNP ID	Exon/Intron	Amino Acid change	Heritability
New allele 1	G*01:01:01:01	chr6:29796949	T	C	rs1464043200	Intron 4	NA	Not detected
New allele 2	G*01:04:01:01	chr6:29797323	C	T	rs144753960	Exon 5	p.Q255Ter	Not detected

* The nucleotides that correspond to the original allele (= the most similar known allele)

Supplementary table 3. Novel SNP associated with HLA-G*01:04:01:01

SNP ID	Linked with	Position	Ref	Alt	4.7KJPN	GnomAD-EAS
rs17875394	G*01:04:01:01	chr6:29795179	G	A	0.1488	0.0855

Supplementary Table 4. Difference of length of T-strech in HLA-G region in shared chromosomes between mother and offspring.

	T mothers			
	17=< NT =< 25	26=< NT =< 35	36=< NT	Total
Average dABS	0.813	1.2	2.8	1.26
Pairs (Total)	16	10	5	31
Matched (dABS= 0)	9	4	1	14
dABS >= 3	2	2	2	6

T mothers: length of HLA-G T streches in shared chroosomes on mother's side.

NT: length of T stretches, dABS: absolute difference of T stretch length between mother and offspring in the shared chromosomes

Supplementary Table 5. Primer sequences used in this study

Primer Name	sequence	mer	Remarks
HLA-G-F3	/5AmMC6/ gcagtcgaacatgtagctgactcaggtcac TGTTCTTTGCAGTTGGCCTTTAATA	55	Amplification of HLA-G containing region (5.2kb)
HLA-G-R4	/5AmMC6/ tggatcacttgtgcaagcatcacatcgtag AGGCTTATGGCTCCAATTATCCA	53	
BC0001_Forward	GGTAG TCAGACGATGCGTCAT gcagtcgaacatgtagctgactcaggtca	50	Barcode addition for PacBio sequencing
BC0002_Reverse	CCATC GCAGAGTCATGTATAG tggatcacttgtgcaagcatcacatcgta	50	
BC0003_Forward	GGTAG TACTAGAGTAGCACTC gcagtcgaacatgtagctgactcaggtca	50	
BC0004_Reverse	CCATC CATGTACTGATACACA tggatcacttgtgcaagcatcacatcgta	50	
BC0005_Forward	GGTAG ACACGCATGACACACT gcagtcgaacatgtagctgactcaggtca	50	
BC0006_Reverse	CCATC GCATATAGTAGAGATC tggatcacttgtgcaagcatcacatcgta	50	
BC0007_Forward	GGTAG ACAGTCTATACTGCTG gcagtcgaacatgtagctgactcaggtca	50	
BC0008_Reverse	CCATC AGATGTAGCACATCAT tggatcacttgtgcaagcatcacatcgta	50	
BC0009_Forward	GGTAG CTGCGTGCTCTACGAC gcagtcgaacatgtagctgactcaggtca	50	
BC0010_Reverse	CCATC AGTCATCGTATCGCGC tggatcacttgtgcaagcatcacatcgta	50	
BC0011_Forward	GGTAG CGCGCTCAGCTGATCG gcagtcgaacatgtagctgactcaggtca	50	
BC0012_Reverse	CCATC TCTGTAGTGCGTGCGC tggatcacttgtgcaagcatcacatcgta	50	
BC0013_Forward	GGTAG ACACTGACGTCGCGAC gcagtcgaacatgtagctgactcaggtca	50	
BC0014_Reverse	CCATC TATACGTATATAGACG tggatcacttgtgcaagcatcacatcgta	50	
BC0015_Forward	GGTAG ATAGAGACTCAGAGCT gcagtcgaacatgtagctgactcaggtca	50	
BC0016_Reverse	CCATC TCTACTCTCGCATCTA tggatcacttgtgcaagcatcacatcgta	50	
HLA-G_S_E2-I2F	CCGCGGTCCTGGTTCTAAA	19	Amplification and sequence primer for sanger sequence
HLA-G_S_E2-I2R1	CATGGAGGTGGGGGTCGTG	19	
HLA-G_S_E2-I2R2	ATCCACTGGAGGGTGTGAGAA	21	
HLA-G_S_E4F	GCCCAGACCCTCTACCTG	18	
HLA-G_S_E4R	AGCATCTCCTTCCCGTTCTC	20	
HLA-G_S_I4F	GCCTCCCTGATCTCCTGTAG	20	
HLA-G_S_I4R	GTTCTAGTCTCTGAGCGGGG	20	
HLA-G_S_E5F	CCCCGCTCAGAGACTAGAAC	20	
HLA-G_S_E5R	CGTGTATCTCTGCTCCTCTCC	21	
HLA-G_S_I5-E6F	GCAGAGATACACGTGCCATG	20	
HLA-G_S_I5-E6R	CACTTCTACCTGGGGCTTGA	20	
HLA-G_S_I6F	GAGCTCAGGTAAGGAAGGGG	20	
HLA-G_S_I6R	GTCTTGGACCCTCGAGAAGT	20	
HLA-G_S_I7F	GGACATTTTCTTCCCACAGA	20	
HLA-G_S_I7R	GGTGACACTTTAAACAGCCCA	21	
HLA-G_S_US1F	CCTGTCTTTACACCTACAATCCC	23	
HLA-G_S_US1R	CACAGGTTAGGAGAAGGAGGAG	22	
HLA-G_S_US2F	AGGGGTTACCAAGGTTATGCTAC	23	
HLA-G_S_US2R	ACACAGGTTAGGAGAAGGAGGAG	23	
HLA-G_S_DS1F	CAGGGCTCTAATGTGTCTCTCA	22	
HLA-G_S_DS1R	AACCCATCAATCTCTCTTGGAA	22	
HLA-G_S_DS2F	CAGTGGTGCAATCTCAACTCAT	22	
HLA-G_S_DS2R	CTCAGGGCAGGAAGAAGAGTA	21	
HLA-G_S_US_01040101_F	TCAGGGGTTACCAAGATTATGCT	23	
HLA-G_S_US_01040101_R	CCAAGCGTTCTGTCTCAGTGTCT	23	

Supplementary Figure 1

Comparison of variant allele frequencies in the in the 3.2 kbp HLA-G core region between the present study and publicly available databases. Panel A and B. Scatter plot of the variant allele frequencies between the present data and 4.7KJPN or GnomAD EAS. Vertical axes indicate the variant allele frequencies in the HLA-G region in this study. Horizontal axes in the Panels B and C indicate the corresponding variant allele frequencies in 4.7KJPN or GnomAD EAS, respectively. The regression lines are shown with broken lines and R-square values are indicated inside the graphs.

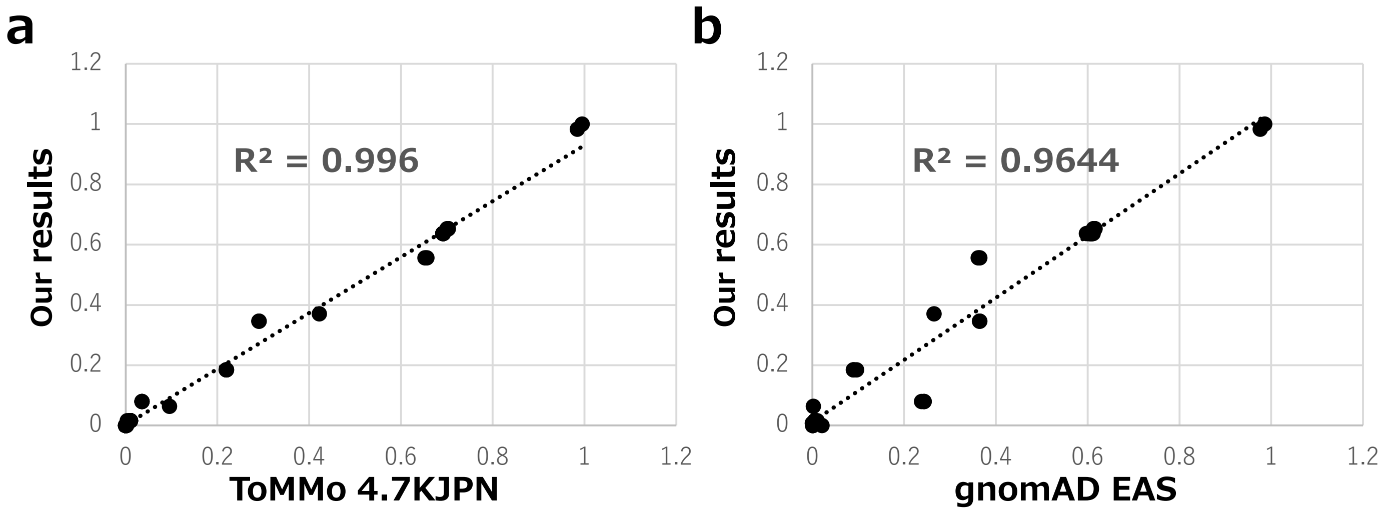

Supplementary Figure 2

Length of T stretches at the HLA-G downstream inherited from mother to offspring. The vertical axis indicates the number of T stretches. Horizontal axis indicates the HLA-G haplotypes divided by the mother's status of HDP onset. Boxes are distribution of the T stretches (from 25% to 75%) and horizontal lines and X marks in the boxes indicate the mean numbers and the medians of T stretches of each haplotype categories, respectively. The minimum and maximum are indicated with whiskers.

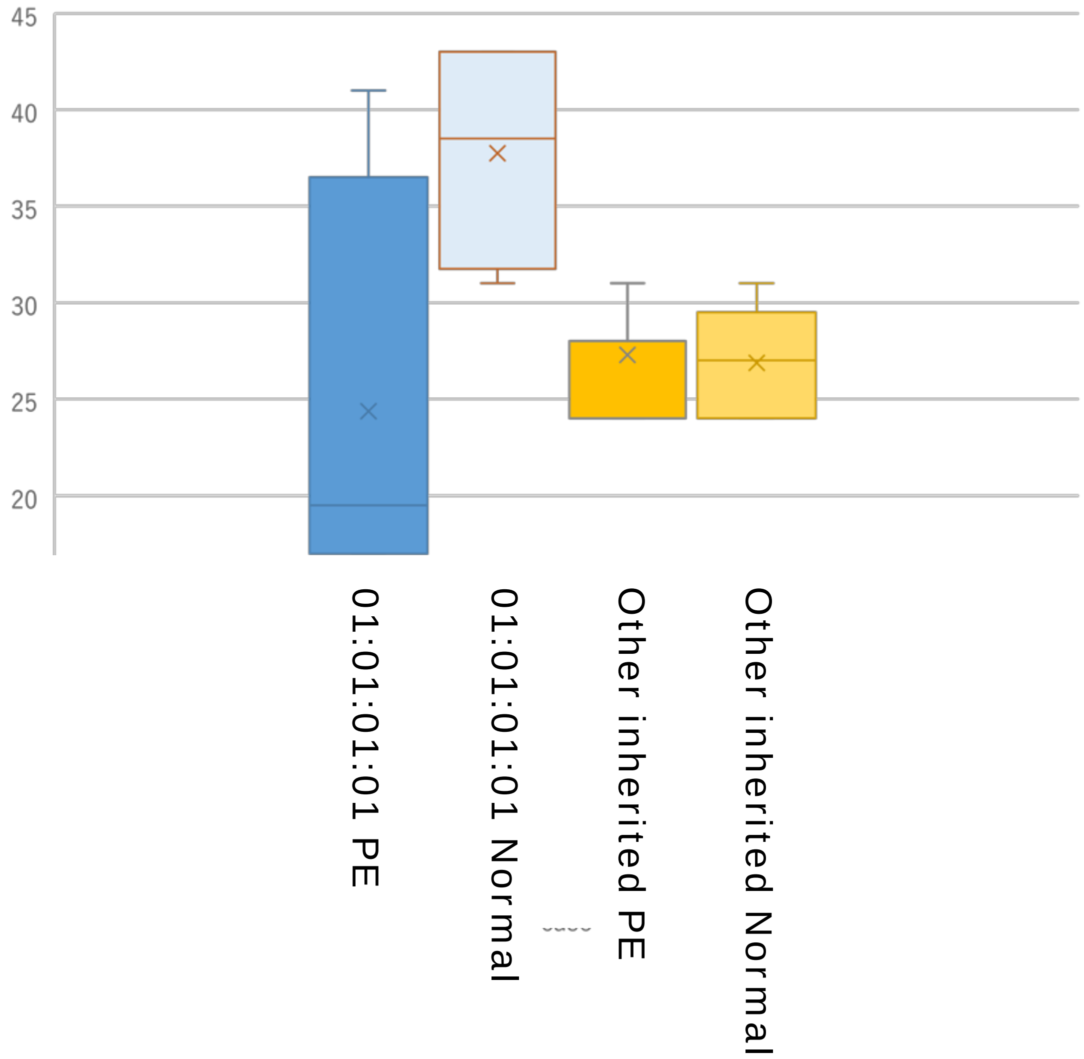

Supplement: Supplementary file 1 — Supplementary Information. [file 41598_2020_77081_MOESM1_ESM.pdf]
